# The longitudinal association between multiple job holding and long-term sickness absence among Danish employees: an explorative study using register-based data

**DOI:** 10.1007/s00420-017-1243-x

**Published:** 2017-07-01

**Authors:** Stef Bouwhuis, Anne Helene Garde, Goedele A. Geuskens, Cécile R. L. Boot, Paulien M. Bongers, Allard J. van der Beek

**Affiliations:** 1 Department of Public and Occupational Health, Amsterdam Public Health research institute, VU University Medical Center, Van der Boechorststraat 7, 1081 BT Amsterdam, The Netherlands; 2 Netherlands Organisation for Applied Scientific Research TNO, Schipholweg 77-89, 2316 ZL Leiden, The Netherlands; 3 Body@Work, Research Center on Physical Activity, Work and Health, TNO-VU/VUmc, Amsterdam, The Netherlands; 4 Department of Psychosocial Work Environment, National Research Center for the Working Environment, Lersø Parkallé 105, DK-2100 Copenhagen Ø Copenhagen, Denmark

**Keywords:** Multiple job holding, Long-term sickness absence, Dual job holding, Register data, Cohort

## Abstract

**Purpose:**

Multiple job holding (MJH) is common in many countries, but little is known about its (health) consequences. Our aim is to explore the longitudinal association between MJH and long-term sickness absence (LTSA) among Danish employees.

**Methods:**

We included employees (*N* = 8968) who participated in the Danish Work Environment Cohort Study (DWECS), based on a representative sample of the Danish working population. Three dichotomous independent variables were created: MJH in general, combination MJH (i.e. second job as employee) and hybrid MJH (i.e. self-employed in second job). LTSA (≥5 weeks) was measured using the Danish Register for Evaluation of Marginalization during 78 weeks of follow-up. Potential confounders included demographics, health, and work characteristics. Logistic regression analyses were performed to study whether LTSA was associated with MJH in general, combination MJH, and hybrid MJH. Interaction effects for gender, age, total working hours per week (≤37 or >37 h a week), and shift work were tested.

**Results:**

In total, 11.7% (*N* = 1048) of the respondents reported having multiple jobs and 7.6% (*N* = 678) experienced LTSA during follow-up. After adjustment for confounders, no significant association between LTSA and MJH in general (OR = 0.82), combination MJH (OR = 0.81), or hybrid MJH (OR = 0.83) was found. Among employees working more than 37 h per week, combination MJH was associated with a higher likelihood of LTSA (OR = 1.50).

**Conclusions:**

We did not find evidence for an increased likelihood of LTSA among multiple job holders. Future research should study the likelihood of LTSA among subgroups of multiple job holders, e.g. those working long hours.

## Introduction

Long-term sickness absence (LTSA) has a large personal, organisational, and societal impact. On a personal level, it may negatively affect sustainable employability, as it is a risk factor for job termination (Virtanen et al. 2006) and not returning to the labour market (Høgelund et al. 2003). Also, it increases the likelihood of social exclusion (Burr et al. 2011). At an organisational and societal level, LTSA accounts for three quarters of the total sickness absence costs (Andersen et al. 2012) and reduces the supply of labour on the short and long term (Burr et al. 2011; Høgelund et al. 2003).

Previous research on LTSA has shown that it is predicted by health, health behaviour, and work characteristics. For instance, general self-reported health has been found to predict LTSA among women (Peterson et al. 2011). Also, studies have shown that LTSA is predicted by burn-out (Borritz et al. 2010; Peterson et al. 2011), disturbed sleep and fatigue (Akerstedt et al. 2007), (severe) depressive symptoms (Bültmann et al. 2006; Peterson et al. 2011), chronic health complaints (Sterud 2014), and being a heavy smoker or ex-smoker (Christensen et al. 2007). Regarding work characteristics, physical demands, such as working mainly standing or squatting and lifting or carrying loads have been shown to predict LTSA (Sterud 2014; Andersen et al. 2016; Lund et al. 2006), as well as psychosocial demands, such as role conflict, poor management quality, high emotional demands, bullying, and low rewards (Clausen et al. 2012; Lund et al. 2005; Melchior et al. 2003; Nielsen et al. 2013; Ortega et al. 2011; Aagestad et al. 2014). Also, shift work predicted sickness absence longer than two weeks in a study among female carers of the elderly (Tüchsen et al. 2008). Recent studies found that long working hours were associated with fewer sickness absence spells, but more sickness absence days (Lesuffleur et al. 2014) and that long working hours reduced the likelihood of sickness absence (Niedhammer et al. 2013).

Until now, studies on the association between work characteristics and LTSA have focused on characteristics of the main job, and have not addressed multiple job holding (MJH). In many countries, however, MJH is a common phenomenon. For instance, in Denmark, the Netherlands, and Norway between 7% and 10% of employed persons had multiple jobs in 2015 (Eurostat 2015).^1^

Little is known about the health consequences of MJH. Previous research has found mixed results and has suggested a variety of mechanisms linking MJH and health. An Australian qualitative study for instance, found that multiple job holders who work long hours experience time squeeze (Bamberry and Campbell 2012). Studies in the US have found that long working hours can partly explain why multiple job holders have a higher risk at injuries and sleep less (Marucci-Wellman et al. 2014a, b). Multiple job holders may also experience difficulties combining different work schedules and different work roles (Bamberry and Campbell 2012), which may lead to stress. In addition, combining different work schedules may result in working anti-social hours (Marucci-Wellman et al. 2014a), which are associated with work–home conflict (Wirtz et al. 2011) and adverse health outcomes (Allen et al. 2000). Other studies have found a positive association between MJH and (mental) health (Dorenbosch et al. 2015; Jamal et al. 1998). This may be explained by increased job pleasure and self-efficacy resulting from increased task variety and the acquisition of diverse skills.

The inconclusive results of earlier studies on the association between MJH and health may be a result of diversity among multiple job holders (Bamberry and Campbell 2012). This diversity can result in distinct health effects among multiple job holders. A recent Dutch cross-sectional study, for instance, found that reasons for MJH modified the association between MJH and burn-out (Dorenbosch et al. 2015). The prevalence of reasons for MJH may differ between countries with different social security systems and may change over time, explaining inconclusive results of previous research.

The present study aims to explore the association between MJH and LTSA to increase our knowledge on the health consequences of MJH. In line with Kivimaki, we consider LTSA as a global indicator of health (Kivimaki et al. 2003). Previous research has suggested that the association between MJH and health is different for employees with different reasons for MJH (Dorenbosch et al. 2015). We expect that employees with a second job as an employee may have different reasons for MJH than employees who are self-employed in their second job, because workers often have specific reasons to choose self-employment, such as a desire for independence (Taylor 1996). To account for the diversity among multiple job holders, we will distinguish between combination MJH (multiple jobs as an employee) and hybrid MJH (job as an employee as well as being self-employed).

## Methods

### Study population

**Fig. 1:**
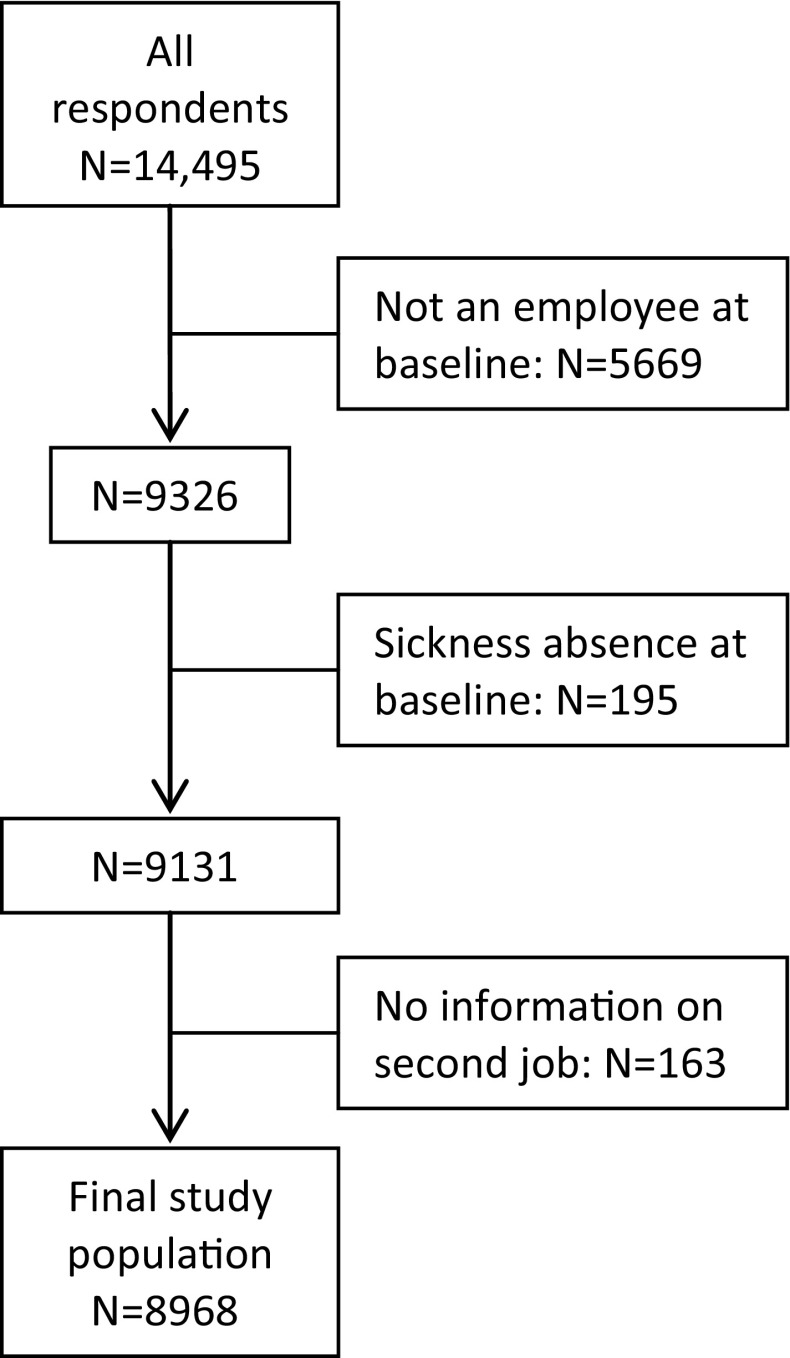
Definition of the study population

The study population consisted of persons who participated in the 2010 wave of the Danish Work Environment Cohort Study (DWECS). In September 2010, a representative sample of 30,000 employees, self-employed and unemployed persons aged 18–59 was extracted from Statistics Denmark and invited to participate in the 2010 wave of DWECS. In total, 14,495 persons returned the questionnaire, either online or on paper. Non-response analyses showed that response was relatively low among men, persons with a short education and younger people (NRCWE 2011). In the present study, we excluded respondents who reported not being an employee at baseline (*N* = 5669), who received a social benefit for sickness absence at baseline (*N* = 195), or who did not report whether they had a second job (*N* = 163). In total, 8968 respondents were included (see Fig. 1).

### Determinant

MJH was measured using one question in DWECS, about having a second job. Possible answers were: ‘yes, as a self-employed person’; ‘yes, as a helping spouse (self-employed)’; ‘yes, as an employee’; and ‘no’. Multiple answers were not allowed. This variable was dichotomized (yes/no). Variables for combination MJH (‘yes as an employee’ versus ‘no’) and hybrid MJH (‘yes as a self-employed person’ and ‘yes as a helping spouse (self-employed)’ versus ‘no’) were also created.

### Outcome

LTSA was defined as receiving sickness benefits for a period of five consecutive weeks or longer, because during the study, according to Danish regulations, employers were entitled to compensation of wages paid to sick-listed workers after a period of four weeks. Consequently, registration of sickness absence started after four weeks. A dichotomous variable was created using data from the Danish Register for Evaluation of Marginalization (DREAM). DREAM contains register-based information on social transfer payments in Denmark, such as sickness absence compensation, and unemployment benefits, on a weekly basis (Hjollund et al. 2007). Follow-up started the week after the respondent filled out the DWECS questionnaire and lasted for 78 weeks (1.5 years). Respondents who died, emigrated, or received pension or ‘cash benefits’ (an unemployment benefit provided by the state alongside which it is not possible to receive sickness benefits) (*N* = 456) were excluded from the week this event occurred, because they were no longer at risk for LTSA. As a result, the average follow-up duration was 76 weeks. Because we used register-based data, information on LTSA was available during the entire follow-up period for all respondents.

### Covariates and confounders

The selection of potential confounders was based on previous research on MJH and LTSA. We included demographic factors, health-related factors, and work-related factors, which were all measured using the DWECS questionnaire, as potential confounders. Regarding demographics, age and gender were included. Both have been shown to be related to LTSA (Burr et al. 2011). Regarding health-related variables, general health and mental health have been shown to be predictors of LTSA (Andrea et al. 2003; Bültmann et al. 2006; Knudsen et al. 2013). Therefore, we included a question on general self-perceived health and the major depression inventory (Olsen et al. 2003). Further, we included several measures of health behaviour, as previous research shows they predict LTSA (Burr et al. 2011; Christensen et al. 2007). Body mass index (BMI = kg/m^2^) was included [underweight and normal weight (up to 25), overweight (25–30), and obese (more than 30)], as well as smoking (never smoked, former smoker, current smoker) and leisure time physical activity (0–2, 2–4 h, more than 4 h of light or moderate physical activity, or more than 4 h of heavy physical activity per week).

To adjust for work-related factors, we included a variable on total working hours per week including overtime, and a dichotomous variable on shift work (permanent day time work versus permanent evening or night work, shift work with/without night work, or other). Both working hours and shift work are predictors of sickness absence (Lesuffleur et al. 2014), and working hours are associated with MJH (Hipple 2010). We also included a scale to measure lifting loads manually, as previous research shows physical work demands are strongly related to LTSA (Burr et al. 2011; Andersen et al. 2016). The selection of psychosocial work factors was also based on previous research, which shows that emotional demands, low rewards, various forms of bullying, and a lack of supportive colleagues and/or management styles predict LTSA (Burr et al. 2011; Aagestad et al. 2014; Lesuffleur et al. 2014; Andrea et al. 2003). We included scales on quantitative demands, emotional demands, social support from colleagues, and social support from the supervisor from Copenhagen Psychosocial Questionnaire (COPSOQ) (Kristensen et al. 2005), and person-related negative behaviour from the Negative Acts Questionnaire (Einarsen and Raknes 1997). Also, we measured quality of management, rewards, work-family conflict, and perceived job insecurity.

### Analyses

Logistic regression analyses were performed to study the association between MJH and LTSA. Separate but identical analyses were performed for MJH in general, combination MJH and hybrid MJH. First, we performed univariable analyses to study the crude associations between MJH and LTSA. Second, we constructed fully adjusted models by entering all potential confounders in the model. This method was chosen to allow comparison between the model for MJH in general, combination MJH, and hybrid MJH. Third, we selected potential confounders per domain (demographics, health, and work characteristic) using backward elimination. We entered all potential confounders per domain in the model and then removed them one by one. The potential confounder that, when removed, caused the smallest change of the coefficient of MJH, was permanently eliminated. This was continued until removing any of the remaining potential confounders changed the coefficient by more than 10%. Subsequently, the same procedure was applied using the remaining potential confounders from all domains together. We chose this procedure for adjusting because it only includes confounders that actually influence the relation between MJH and LTSA, and accounts for joint confounding. In sensitivity analyses, respondents who died, emigrated, or received pension or cash benefits (*N* = 456) were excluded.

To study whether the relation between MJH and LTSA differed by gender, age (<45 years; ≥45 years), total working hours (≤37 h per week; >37 h per week)(Eurofound 2009), or shift work (yes/no), we added interaction terms to the crude models. If an interaction term was significant (*p* < 0.05), stratified analyses were performed. We only performed unadjusted stratified analyses, because of limited statistical power.

## Results

Table 1 shows the characteristics of the study population. In total, 11.7% (*N* = 1048) of the respondents reported having a second job, 60.6% (*N* = 635) of whom had a second job as an employee, and 39.4% (*N* = 415) of whom were self-employed in their second job. Further, 7.6% (*N* = 678) of respondents experienced LTSA during follow-up. LTSA occurred among 7.7% of the single job holders (*N* = 610) and among 6.5% respondents with a second job (*N* = 68). Among respondents who were employee in their second job 6.9% (*n* = 44) experienced LTSA and among respondents who were self-employed in the second job 5.6% (*N* = 24) experienced LTSA.

**Table 1:** Characteristics of the study population (*N* = 8968)

	Single job holding (SJH) *N* = 7920	Multiple job holding (MJH) *N* = 1048	Combination MJH *N* = 635	Hybrid MJH *N* = 413
Demographics				
Gender				
Female	53.7%	46.6%^a^	52.0%	38.3%^a,b^
Age	44.9 (10.5)	44.0 (11.5)^a^	42.7 (12.4)^a^	46.0 (9.6)^a,b^
Age (dichotomous)				
<45 years	45.7%	46.9%	50.7%	41.2%
≥45 years	54.3%	53.1%	49.3%	58.8%
Health				
General self-reported health (0–4)	3.1 (0.8)	3.1 (0.8)^a^	3.1 (0.8)	3.2 (0.8)^a,b^
Depressive symptoms (0–100)	6.5 (6.3)	6.8 (7.2)	7.2 (7.6)^a^	6.1 (6.6)^b^
Body Mass Index (BMI)				
Underweight/normal weight	52.2%	50.7%	52.1%	48.5%^a^
Overweight	34.7%	37.2%	34.9%	40.6%
Obese	13.1%	12.2%	13.0%	10.9%
Health behaviour				
Smoking				
Never smoked	47.4%	52.9%^a^	50.7%	56.3%^a,b^
Smoked in the past	30.2%	26.5%	25.9%	27.5%
Current smoker	22.4%	20.5%	23.4%	16.2%
Leisure time physical activity				
<2 h per week	24.9%	26.5%^a^	26.5%^a^	26.6%
2–4 h per week	39.1%	33.9%	33.4%	34.8%
>4 h per week (low medium)	32.2%	33.8%	32.9%	35.3%
>4 h per week (heavy)	3.7%	5.7%	7.3%	3.3%
Work characteristics				
Working hours				
Total working hours	37.9 (7.3)	45.5 (12.3)^a^	45.0 (13.6)^a^	46.0 (10.4)^a^
Total working hours (dichotomous)				
≤37 h	63.5%	18.9%^a^	20.5%^a^	14.3%^a,b^
>37 h	36.5%	82.1%	79.5%	85.7%
Shift work				
Yes	19.1%	26.6%^a^	31.2%^a^	19.7%^b^
Physical load				
Lifting loads index (0–100)	15.3 (19.4)	16.4 (19.8)	17.6 (20.4)^a^	14.5 (18.8)^b^
Psychosocial factors				
Quantitative demands (0–100)	45.0 (19.7)	45.0 (19.9)	42.5 (20.4)^a^	48.8 (18.4)^a,b^
Emotional demands (0–100)	45.0 (24.8)	48.6 (24.8)^a^	49.7 (24.6)^a^	46.9 (24.9)
Social support colleagues (0–100)	73.1 (21.1)	71.7 (21.9)	71.7 (22.8)	71.8 (20.4)
Social support management (0–100)	69.6 (24.6)	68.0 (25.7)	67.6 (27.0)	68.6 (24.0)
Quality of management (0–100)	53.0 (22.3)	52.9 (21.9)	52.3 (21.9)	53.8 (21.8)
Person-related negative behaviour (0–100)	6.3 (12.5)	8.0 (13.8)^a^	8.6 (14.3)^a^	7.0 (12.9)
Rewards (0–100)	56.8 (17.6)	58.6 (18.0)^a^	57.6 (18.2)	60.2 (17.6)^a,b^
Work-family conflict (0–100)	31.4 (25.2)	32.4 (25.6)	31.5 (25.3)	33.8 (25.9)
Perceived job insecurity				
Yes	31.1%	28.0%^a^	32.0%	21.8%^a,b^
Outcome				
LTSA	7.7%	6.5%	6.9%	5.8%

^a^Differs significantly from single job holders

^b^Hybrid MJH differs significantly from combination MJH

After adjusting for demographics, health, and work characteristics, we found no statistically significant relation between MJH, combination MJH, hybrid MJH, and LTSA (see Table 2). All odds ratios (ORs) were below one, and the ORs for combination MJH and hybrid MJH were similar. The sensitivity analyses showed ORs similar to those in the main analyses (data not shown).

**Table 2:** The longitudinal association between MJH and LTSA

Model	Multiple job holding (MJH)		Combination MJH		Hybrid MJH	
	OR	95% CI	OR	95% CI	OR	95% CI
Crude	0.83	0.64–1.08	0.89	0.65–1.23	0.74	0.49–1.13
Fully adjusted^a^	0.82	0.58–1.17	0.81	0.52–1.25	0.83	0.48–1.44
Adjusted (backward elimination)	0.80^b^	0.57–1.11	0.78^c^	0.52–1.19	0.82^d^	0.51–1.34

^a^Adjusted for all confounders

^b^Adjusted for: total working hours, shift work, lifting loads, general health, and leisure time physical activity

^c^Adjusted for: age, total working hours, shift work, lifting loads, emotional demands, quantitative demands, work-family conflict, and leisure time physical activity

^d^Adjusted for: total working hours, shift work, work-family conflict, general health, smoking, and leisure time physical activity

The interaction terms for working hours and MJH in general and combination MJH were statistically significant. Therefore, we stratified the analyses of the association between these types of MJH and LTSA by total working hours. Table 3 shows that among employees who worked 37 h per week or less, MJH in general as well as combination MJH was associated with a lower likelihood of LTSA in a crude analysis. Among employees who worked more than 37 h per week, combination MJH was associated with a higher likelihood of LTSA.

**Table 3:** The longitudinal association between MJH and LTSA, stratified by weekly working hours (crude analyses)

Model	Multiple job holding (MJH)				Combination MJH			
	37 h per week or less		More than 37 h per week		37 h per week or less		More than 37 h per week	
	OR	95% CI	OR	95% CI	OR	95% CI	OR	95% CI
Crude	0.26^a^	0.10–0.71	1.29	0.93–1.77	0.20^a^	0.05–0.80	1.50^a^	1.03–2.20

^a^OR is statistically significant, *p* < 0.05

## Discussion

The aim of this study was to explore the association between MJH and LTSA. Overall, MJH, combination MJH, and hybrid MJH were not significantly associated with LTSA. The direction of the ORs suggests that among Danish employees there are no indications for a higher likelihood of LTSA among multiple job holders. However, among employees who worked more than 37 h per week, combination MJH might be associated with a higher likelihood of LTSA. We also found that, in fully adjusted analyses, ORs were similar for combination MJH and hybrid MJH. This suggests that any differences between combination MJH and hybrid MJH regarding their association with LTSA are explained by differences in demographics, health, and work characteristics between these types of MJH.

Previous research from the US showed that MJH may adversely affect health and result in more injuries (Marucci-Wellman et al. 2014b, 2016). The present study did not find that MJH was associated with a higher likelihood of LTSA. Three reasons may explain these contrasting findings. Firstly, in the present study, a different outcome measure was examined compared to previous studies on the association between MJH and health. Although LTSA is an indicator of health (Kivimaki et al. 2003) and it is widely used as such, differences may exist. For instance, it is possible that not all adverse health effects of MJH found in previous research result in LTSA.

Secondly, multiple job holders are a heterogeneous group of employees, regarding (demographic) background and reasons for MJH (Bamberry and Campbell 2012). This heterogeneity may also be reflected in different health effects of MJH among different subgroups of multiple job holders (Dorenbosch et al. 2015). Studying a global health indicator, such as LTSA, without looking at health effects for different subgroups of multiple job holders, may result in non-significant findings regarding the association between MJH and health. The present study revealed no differences between combination MJH and hybrid MJH regarding the likelihood of LTSA, but other relevant subgroups may exist (e.g. multiple job holders working longer hours). Future research should study whether other subgroups of multiple job holders can be identified and examine whether health effects of MJH differ between these subgroups.

The heterogeneity among multiple job holders may also affect the relation between health and LTSA. For some workers, having multiple jobs is a financial necessity. For this subgroup of multiple job holders, adverse health effects of MJH may not be associated with a higher likelihood of LTSA but with a higher likelihood of presenteeism due to fear of losing their job. Future research should address not only whether health effects of MJH are different among different subgroups but also whether these subgroups react differently to the health effects of MJH.

Thirdly, socioeconomic differences and differences in social security systems between the US and Denmark may explain why we did not find a higher likelihood of LTSA among Danish multiple job holders. For instance, it is possible that because of socioeconomic differences between these two countries, in the US more workers have multiple jobs due to financial necessity than in Denmark. This in turn may result in different health effects of MJH in these countries, because reasons for MJH seem to modify its relation to health (Dorenbosch et al. 2015). However, very little is known about how socioeconomic differences and reasons for MJH modify the health effects of MJH. Future research should study whether the health effects of MJH are different for multiple job holders with different socioeconomic backgrounds and with different reasons for MJH.

That we did not find an increased likelihood of LTSA among multiple job holders may also be explained by a healthy worker effect (McMichael 1976). It is possible that good health is a prerequisite for having multiple jobs and working long hours. We found that, at baseline, only those who are self-employed in their second job have better health than employees with one job. However, based on our analyses we cannot conclude whether a health worker effect among multiple job holders exists and whether it is stronger than a healthy worker effect among single job holders. More longitudinal research on the relation between MJH and health is needed to determine whether health is a determinant and/or an effect of MJH.

The finding that those who have multiple jobs as an employee and work more than 37 h per week have a higher risk of LTSA is not in line with previous research on working hours and sickness absence. A recent French study found that employees working long hours experienced fewer sickness absence spells (Lesuffleur et al. 2014). An explanation of these contradicting findings may be that having multiple jobs is an extra burden for those who work long hours, increasing their risk of sickness absence. More research on how the combination of MJH and long working hours influences health is necessary.

A strength of this study is the use of register-based data, which meant that for all respondents information on LTSA was available during the entire follow-up period. Also, the way LTSA was measured warrants high reliability, as employers have a strong financial incentive to register ill employees. A further strength is that we distinguished between combination MJH and hybrid MJH to account for heterogeneity among multiple job holders. A first weakness is that the statistical power of the study was limited, because of the low incidence of LTSA. This limited power is reflected by the relatively wide confidence intervals of the ORs. Secondly, it is possible that some health effects of MJH only occur after an employee has had multiple jobs for a longer period of time. However, we only had information on MJH from one measurement. Therefore, we were not able to determine MJH-history of respondents. Thirdly, the measurement of MJH as well as all potential confounders was based on self-reported data. Regarding the question on MJH, respondents who had more than two jobs were not able to indicate this. Therefore, it is possible that misclassification occurred as employees who had a second job as an employee and were self-employed in a third job were mistakenly classified as combination MJH. Fourthly, we did not have data on socioeconomic status (SES) of respondents. However, a Danish study found no significant association between SES and LTSA after adjusting for demographics, physical and psychosocial work environment, family status, and health behaviour (Christensen et al. 2008). Because we adjusted for a wide set of confounders, e.g. physical and psychosocial work demands and health behaviour, we are confident that including SES as a potential confounder would not have substantially changed our results. Fifthly, previous research has suggested that the association between MJH and health may differ between employees with different reasons for MJH (Dorenbosch et al. 2015). As we did not have information on these reasons, we were not able to take this into account in our analyses. Sixthly, the interaction terms were only tested in crude analyses, and not in adjusted analyses, because of limited statistical power.

In conclusion, this study adds to our knowledge that MJH is not associated with an overall increased likelihood of LTSA among Danish employees. However, we found indications for an increased likelihood of LTSA among specific subgroups of multiple job holders, e.g. those working more than 37 h per week and having multiple jobs as an employee. However, little is known about which subgroups of MJH exist and how MJH is related to health and LTSA among those subgroups. Future research is recommended to study which subgroups of multiple job holders exist and whether health effects of MJH differ between these subgroups.

## Footnotes

1 Number of employed persons with a second job divided by total number of employed persons.
